# Glycosylation at 11Asn on hemagglutinin of H5N1 influenza virus contributes to its biological characteristics

**DOI:** 10.1186/s13567-017-0484-8

**Published:** 2017-11-21

**Authors:** Yuncong Yin, Xiaojian Zhang, Yiyi Qiao, Xiao Wang, Yangyang Su, Sujuan Chen, Tao Qin, Daxin Peng, Xiufan Liu

**Affiliations:** 1 College of Veterinary Medicine, Yangzhou University, Yangzhou, 225009 Jiangsu China; 2 Jiangsu Co-Innovation Center for the Prevention and Control of Important Animal Infectious Disease and Zoonoses, Yangzhou, 225009 Jiangsu China; 3 Jiangsu Research Centre of Engineering and Technology for Prevention and Control of Poultry Disease, Yangzhou, 225009 Jiangsu China

## Abstract

A stem glycosylation site of hemagglutinin (HA) is important to the stability of the HA trimmer. A previous study shows that the stem 10/11 overlap glycosylation site of the H5 subtype avian influenza virus may influence the cleavage of HA, whereas the exact site and its effect on virulence remain unclear. In this study, site-directed mutagenesis was used to generate single or double mutant rSY-Δ10(10NNAT), rSY-Δ11(10NNSA), and rSY-Δ10/11(10NNAA) of the overlapping glycosylation site (10NNST) on the HA of A/Mallard/Huadong/S/2005(SY). By using Western blot analysis, we show that both rSY-Δ11 and rSY-Δ10/11 mutant viruses had significant delay on HA cleavage and a reduced HA molecular mass compared to the wild-type virus rSY, while the rSY-Δ10 mutant virus exhibited a similar HA molecular mass to that of the wild-type virus rSY. Interestingly, both rSY-Δ11 and rSY-Δ10/11 mutant viruses reverted their glycosylation sites at 11N after passage, indicating that 11N is a true and critical glycosylation site. Compared to the wild-type virus rSY, rSY-Δ11 and rSY-Δ10/11 mutant viruses had decreased growth rates, reduced thermo- and pH-stability, decreased pathogenicity, and limited systemic spread. Therefore, our study suggests that the 11N glycosylation site plays a key role in HA cleavage, structural stability and pathogenicity in H5 subtype avian influenza virus.

## Introduction

H5 subtype avian influenza virus (AIV) infects not only poultry but also mammals worldwide [1–3], thus posing a threat to the poultry industry and to public health [4, 5]. Hemagglutinin (HA), a surface glycoprotein, plays an important role in the influenza life cycle [4, 6]. As the avian influenza virus evolves, glycosylation distribution of HA is becoming increasingly complicated [7, 8]. Glycosylation sites function differently depending on their location: the glycan near the antigen epitope may cause immune escape by disturbing antibody recognition [9–11]; the glycan near the cleavage sites may result in virulence reduction due to HA cleavage deficiency [12, 13]; the glycan near the receptor binding site may change its receptor affinity [14, 15]. Stem glycosylation of HA appears conserved, mainly attributed to the stability of the HA trimer [14, 16]. A previous study shows that there is a potential 10/11 glycosylation site overlap on the HA stem of the SY virus, which plays an important role in cleavage [17]. However, the exact glycosylation site remains unclear. In this study, site-direct mutagenesis was used to delete the overlapping glycosylation site, so biological characteristics of the mutants could be determined.

## Materials and methods

All animal studies were approved by the Jiangsu Administrative Committee for Laboratory Animals (Permission Number: SYXKSU-2007-0005) and complied with the Guidelines of Laboratory Animal Welfare and Ethics of Jiangsu Administrative Committee for Laboratory Animals.

### Viruses and cells

Madin–Darby canine kidney (MDCK) cells, human embryonic (293T) cells and chicken embryo fibroblast (CEF) cells were maintained in Dubecco’s modified Eagle’s medium (DMEM) with 10% fetal bovine serum (FBS, Foundation, Gemini) at 37 °C with 5% CO2. AIV A/Mallard/Huadong/S/2005 (SY, H5N1) [18] was propagated in 10-day-old specific-pathogen-free (SPF) embryonic chicken eggs.

### Site-directed mutagenesis, virus rescue and identification

Site-directed mutagenesis of the HA gene of the H5N1 AIV SY strain was performed by overlap-PCR with the primers indicated in Table 1. To delete N-glycosylation sites at 10/11NN, 12Ser and 13Thr were substituted separately or simultaneously with Ala. The modified HA genes were cloned to the pHW2000 vector and confirmed by sequencing [19]. Then, the eight rescue plasmids with or without mutant HA plasmids were co-transfected into a mixture of 293T and MDCK cells using polyjet (SignalGen). The culture mixtures were treated with repeated freeze–thaw at 48 h post-transfection and then inoculated into 10-day-old SPF eggs for amplification of rescue viruses at 37 °C. All rescue viruses were then tested individually for the presence of infectious viruses through a standard hemagglutination assay by 1% chicken red blood cells. The RNA of the rescue viruses were extracted by Trizol (Invitrogen) and amplified by RT-PCR. All viral gene segments were sequenced to ensure the absence of unwanted mutations. Each rescue virus was passaged at least five generations in SPF eggs or chicken embryo cells. To measure the virus titer, the individual virus was serially diluted tenfold from 10^−1^ to 10^−9^, and each dilution (10^−5^–10^−9^) was inoculated into four 10-day-old SPF eggs or CEF cells. The 50% chicken embryo infection dose (EID_50_/mL) and 50% tissue culture infection dose (TCID_50_/mL) were calculated as previously described [20].

**Table 1:** **Mutagenesis primers for the hemagglutinin gene**

Mutation	Direction	Primer sequence (5′-3′)
SY S10A	Forward	CATGCAAACAACGCGACAGA
	Reverse	TCTGTCGCGTTGTTTGCATG
SY T11A	Forward	CATGCAAACAACTCGGCAGA
	Reverse	TCTGCCGAGTTGTTTGCATG
SY ST10/11AA	Forward	CATGCAAACAACGCGGCAGA
	Reverse	TCTGCCGCGTTGTTTGCATG

The substitution nucleotides are underlined.

### Western blot analysis

To analyze the molecular mass of HA protein in the viruses [21], CEF cells were inoculated with the recombinant viruses at a multiplicity of infection (MOI) of 1 and incubated for 1 h at 37 °C with 5% CO2. The infected cells were washed three times with PBS and then fresh DMEM containing 2% FBS was added. At 12 h incubation, the cells were washed with pre-cooled PBS, scraped and lysed with 200 μL of lysis buffer (Thermofisher Scientific) individually on ice for 15 min. Total proteins were collected by centrifugation at 13 000 rpm at 4 °C for 10 min, subjected to 12% SDS-PAGE, and transferred to PVDF membrane. The membrane was blocked in 5% skimmed milk, incubated with mAb SYA9 and polyAb anti-M_1_ mouse serum, and then incubated with horseradish peroxidase-conjugated goat anti-mouse antibodies. The protein bands were developed using a chemiluminescence imaging analysis system. For time-point analysis, the CEF monolayer cells infected with each recombinant virus was taken at 12 h intervals from 12 to 72 h post-infection (hpi) and frozen in −80 °C. All collected cells were subjected to Western blot analysis.

### Virus growth

Monolayer CEF cells were infected with each recombinant virus at an MOI of 1 in DMEM for 1 h. Then cells were washed to remove unbound viruses and fresh DMEM was added. The cells were incubated at 37 °C with 5% CO_2_ and supernatants were sampled every 12 h. After 72 hpi, TCID_50_ on CEF cells were determined for all samples [22].

### Thermostability

Recombinant viruses were divided into nine 60 μL aliquots. All aliquots were exposed to 56 °C and each recombinant group was quickly cooled to 4 °C after 0, 5, 10, 15, 30, 60, 90, 120 and 150 min incubation [23]. The titers of all aliquots were then tested by standard hemagglutination assay with 1% chicken red blood cells. All recombinant viruses were also diluted to the same TCID_50_ and incubated at 37 or 42 °C for 1, 3 and 5 days. The titers of all aliquots were tested by TCID_50_. In addition, methanol-inactivated recombinant viruses were incubated at 37 or 42 °C at a 2-h interval for 18 h. The titers of all samples were determined by hemagglutination assay.

### pH stability

Recombinant viruses were mixed with an equal volume of 100 mM acetate buffer (pH = 4.0 and pH = 5.0), 100 mM phosphate buffer (pH = 6.0), or neutral phosphate buffer (pH = 7.0) [24]. After a 10-min incubation at 37 °C, the titers of all samples were determined by hemagglutination assay.

### IVPI determination in chickens

Six-week-old SPF white leghorn chickens (10 per group) were injected intravenously with 0.1 mL of 1:10 diluted recombinant virus. Chickens were monitored daily for clinical signs of disease for 10 days, and the intravenous pathogenicity indices (IVPI) were calculated according to the OIE recommendation.

### Virulence determination in mice

Eight-week-old BALB/c mice (5 per group) were infected intranasally with 10^4^ or 10^6^ EID_50_ of each virus in 50 μL PBS. The mice were weighed individually and monitored for signs of illness and mortality for 2 weeks. In addition, 8-week-old mice (6 per group) were infected intranasally with 10^4^ EID_50_ of each virus in 50 μL PBS. Three mice from each group were euthanized on days 3 and 6 post-infection, and the lungs, brains, kidneys, spleens, hearts and livers were collected for virus titration [25–27].

### Statistical analysis

The viral titers and antibody titers are expressed as the mean ± standard deviation. Statistical analyses were performed using a Mann–Whitney test. Differences with a *p* value of less than 0.05 were regarded to be statistically significant.

## Results

### Rescue of the mutant viruses

The overlapping glycosylation site at 10/11 in HA was modified by changing the rSY amino acid sequence NNST to NNAT, NNSA or NNAA, and the respective mutants were named rSY-Δ10, rSY-Δ11 and rSY-Δ10/11. All rescued viruses were confirmed by sequence analysis and were without spontaneous mutations in the first generation. However, after passage in SPF chicken embryonic egg or CEF for three generations, rSY-Δ11 reverted from NNSA to NNST and rSY-Δ10/11 from NNAA to NNAT, while no reversion was found for NNAT in rSY-Δ10. Thus, all mutant viruses of the first generations were used for further experimentation except when indicated.

### Western blot analysis

**Figure 1:**
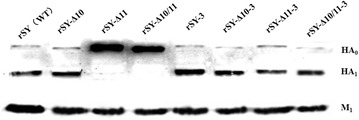
**Western blot analysis of HA**_**1**_ **protein from recombinant virus.** Lysates of CEF infected with H5N1 viruses at an MOI of 1 for 12 h were incubated with mAb SYA9 (anti-HA1 of H5N1) and mouse serum (anti-M1 of H5N1). The bands were visualized by a chemiluminescence imaging analysis system after incubation with peroxidase-conjugated secondary antibodies.

Western blot was used to determine whether the glycosylation site was removed from HA stem protein. As shown in Figure 1, M_1_ proteins were expressed equally in all mutant viruses as well as in wild-type virus rSY, while HA_1_ from the mutant virus rSY-Δ10 showed a similar molecular mass to that of rSY at 12 hpi. However, HA_1_ proteins from the mutant viruses rSY-Δ11 or rSY-Δ10/11 were not detectable, indicating HA_0_ was not lysed at that time. After the third passage, HA_1_ proteins from the mutant viruses of rSY-Δ11-3or rSY-Δ10/11-3 revealed similar molecular masses to that from the rSY, indicating that both mutant viruses rSY-Δ11 and rSY-Δ10/11 recovered cleavage activity of HA_0_.

**Figure 2:**
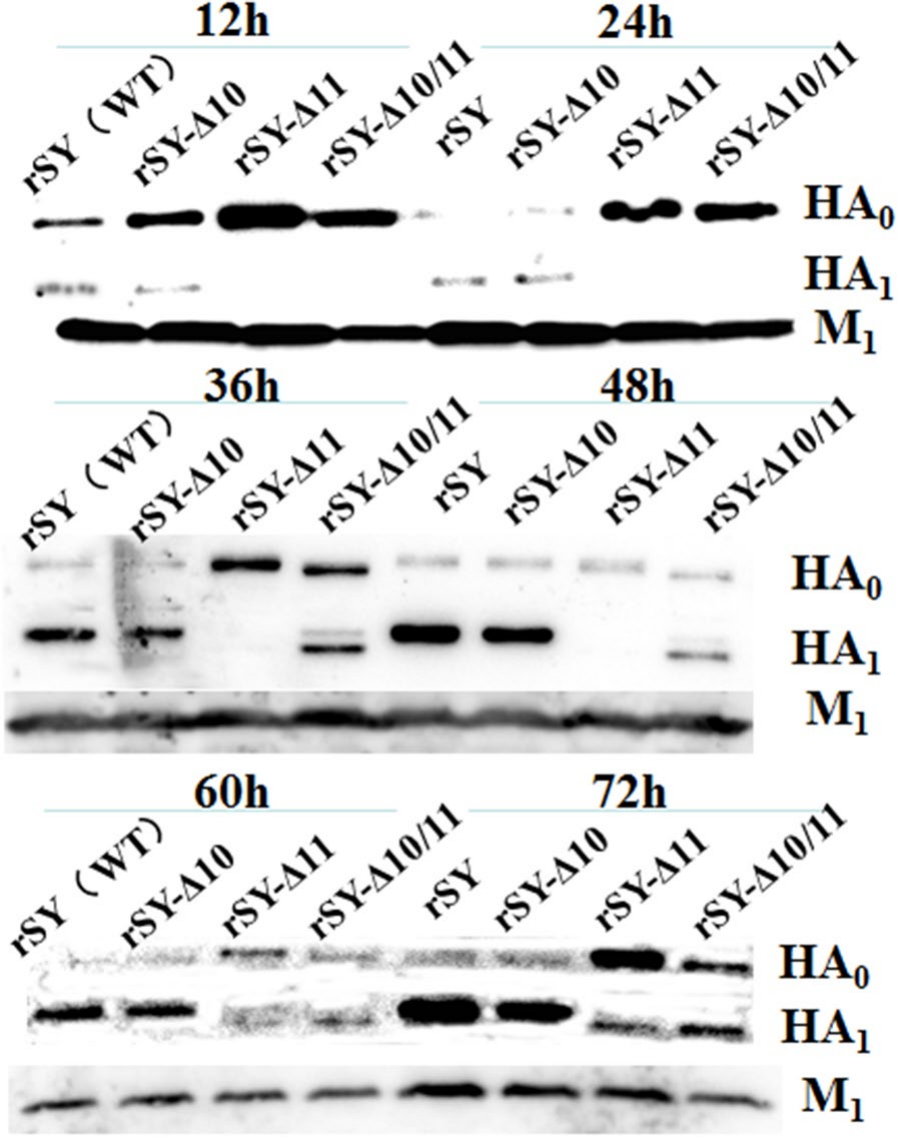
**Detection of HA cleavage.** All mutant viruses infected CEF at an MOI of 1 for 72 h. Samples were taken at 12-h intervals and subjected to Western blot analysis.

Next, HA_1_ proteins were detected at different time points (Figure 2). HA_1_ proteins from the rSY and rSY-Δ10 viruses could be detected as early as 12 hpi, HA_1_ protein from the rSY-Δ10/11 could be detected from 36 hpi, and HA_1_ protein from the rSY-Δ11 could be detected from 60 hpi. HA_1_ protein from mutant viruses rSY-Δ11 and rSY-Δ10/11 both showed reduced molecular masses compared to the rSY and rSY-Δ10 viruses. These data suggest that 11N is the real glycosylation site and its loss may hinder HA protein cleavage in H5N1 AIV.

### Virus growth

**Figure 3:**
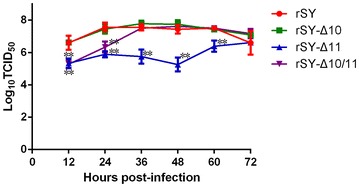
**Growth curve of mutant virus in CEF.** The cell monolayers were infected with mutant viruses at an MOI of 1 for 72 h, and TCID_50_ virus titers were measured in the supernatants at the time points indicated. The error bars represent SD of the means from three independent experiments. The statistical differences in the growth properties between the wild-type virus and mutant viruses was assessed through a Mann–Whitney test (*p < 0.05).

Chicken embryo fibroblast was inoculated with the mutant viruses at an MOI of 1 to determine whether glycosylation site removal would affect virus infection (Figure 3). Compared to wild-type virus rSY, rSY-Δ10 showed the same infectivity and growth tendency. However, the titer of rSY-Δ10/11 was significantly lower than that of rSY within 24 hpi and was similar to that of rSY at 36 hpi. The titer of rSY-Δ11 was significantly lower than that of rSY up to 60 hpi. These data suggest that the 11N glycosylation site deletion may result in low infectivity of H5N1 AIV in CEF.

### Thermal stability of the recombinant viruses

**Figure 4:**
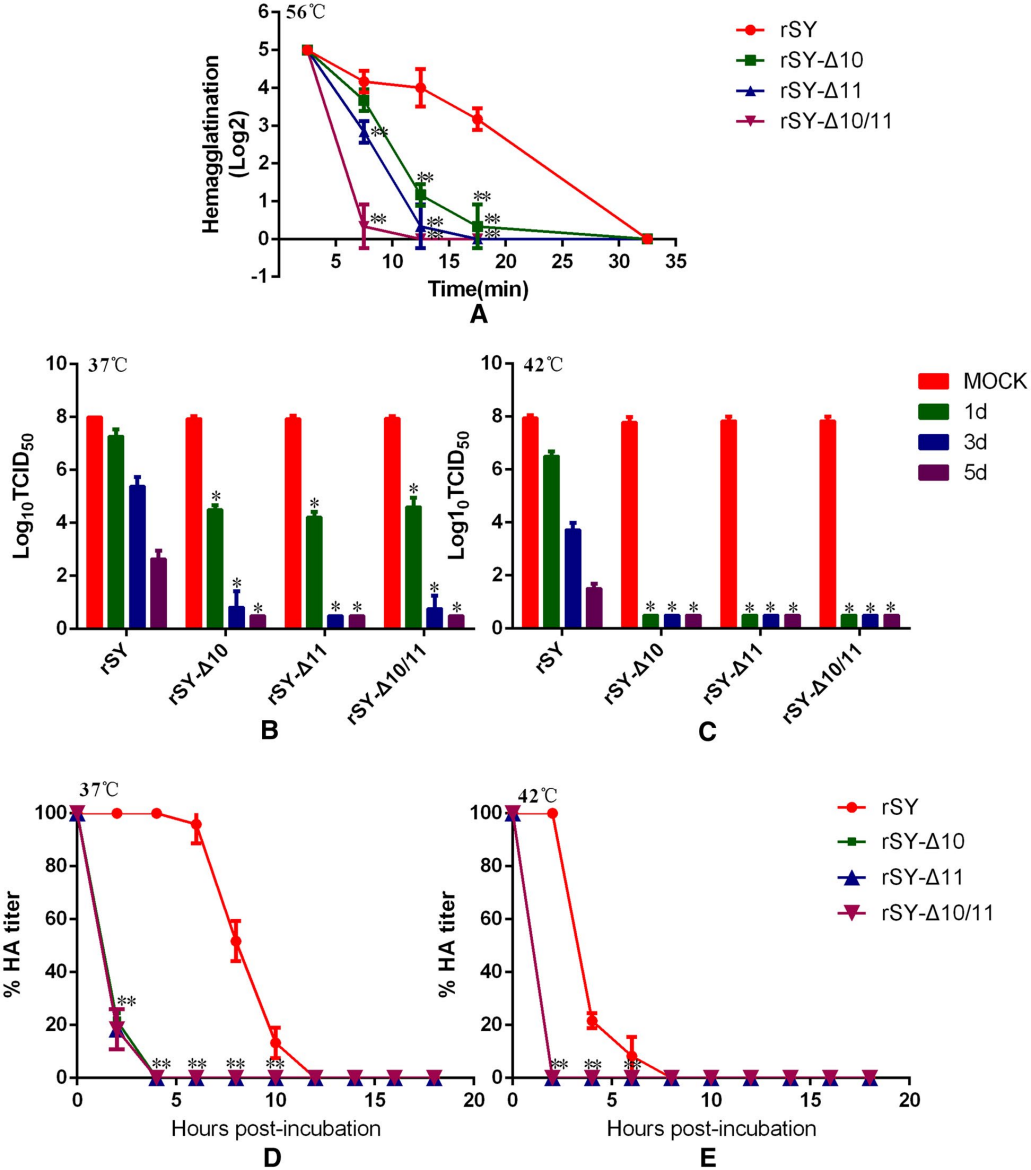
**Thermal stability of the mutant viruses. A** Nine 60-μL aliquots of mutant viruses were exposed to 56 °C for 150 min. All aliquots were tested by hemagglutination assay. The error bars denote SD of the mean of three independent titers at each time point. **B**, **C** The wild-type virus and mutant viruses were incubated at 37 °C (**B**) or 42 °C (**C**) for 5 days. The TCID_50_ titers of the aliquots were determined in CEF cells. **D**, **E** The wild-type virus and mutant viruses were diluted to the same TCID_50_ and inactivated by methanol. Then all viruses were exposed to 37 °C (**D**) or 42 °C (**E**) for 18 h and every aliquot was collected every 2 h. The titers of the aliquots were determined by hemagglutination assay. An asterisk indicates that the titer of the mutant virus was significantly different from those of the wild-type virus at the time points indicated, as determined by the Mann–Whitney test (*p < 0.05, **p < 0.01).

All mutant viruses were treated with three temperatures (56, 42, 37 °C) to measure their thermal stability (Figure 4). All mutant viruses lost their hemagglutination within 20 min after 56 °C incubation, while hemagglutination titer of the wild-type virus rSY remained 3 log2 (Figure 4A). All live mutant viruses as well as inactivated mutant viruses showed the decreased thermal stability after 37 and 42 °C treatment, when compared to the wild-type virus rSY (Figures 4B–E).

### pH stability of the recombinant viruses

**Figure 5:**
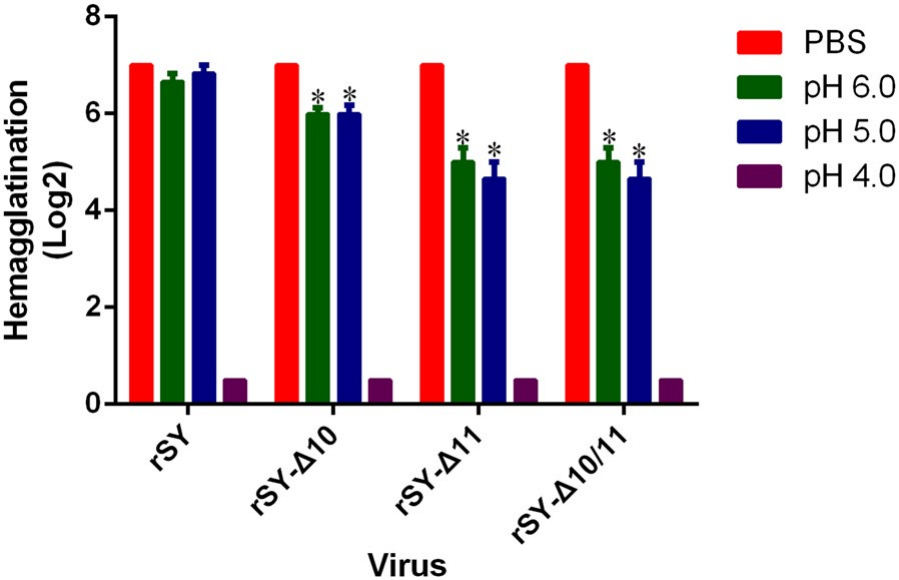
**pH stability of the recombinant virus.** The recombinant viruses were incubated in each buffer at 37 °C for 10 min, and the viral titers were determined by hemagglutination assay. The results are presented as log2 HA titers at the indicated pH conditions. The error bars denote SD of the mean of three independent titers at each time point. The statistical significance of the differences in pH stability between the wild-type virus and mutant viruses was assessed through the Mann–Whitney test (*p < 0.05).

All mutant viruses were exposed to low pH (pH = 4, 5, or 6) to assess their pH stability. The hemagglutination titer of wild-type virus rSY was unchanged when exposed to a pH of 6 or 5. By contrast, the hemagglutination titers of all mutant viruses decreased significantly compared to that of the wild-type virus. All mutant and wild-type viruses lost their hemagglutination when treated with pH = 4 (Figure 5).

### Pathogenicity in chickens

IVPI was used to measure the virulence of four mutant viruses in chickens (Table 2). Chickens injected with rSY and rSY-Δ10 all died within 1 day. The IVPI were up to three in these two groups. Some of the chickens in the rSY-Δ11 and rSY-Δ10/11 groups died 1 day later and their IVPI were 2.89 and 2.86, respectively. These data suggest that modification of 11N-glycosylation may decrease viral virulence in chickens, but the mutant viruses rSY-Δ11 and rSY-Δ10/11 were still highly pathogenic to chickens.

**Table 2:** **Determination of intravenous pathogenicity indexes for the mutant viruses**

Recombinant virus	IVPIs
SY (wild-type)	3
rSY-Δ10	3
rSY-Δ11	2.89
rSY-Δ10/11	2.86

6-week-old SPF white leghorn chickens (10 chickens per group) were inoculated intravenously with 0.1 mL of 1:10 dilution of allantoic fluid containing each virus. Experimental SPF chickens were monitored daily for clinical signs of disease for 10 days, and the intravenous pathogenicity indexes (IVPI) were calculated according to the recommendation of the OIE.

### Pathogenicity in mice

**Figure 6:**
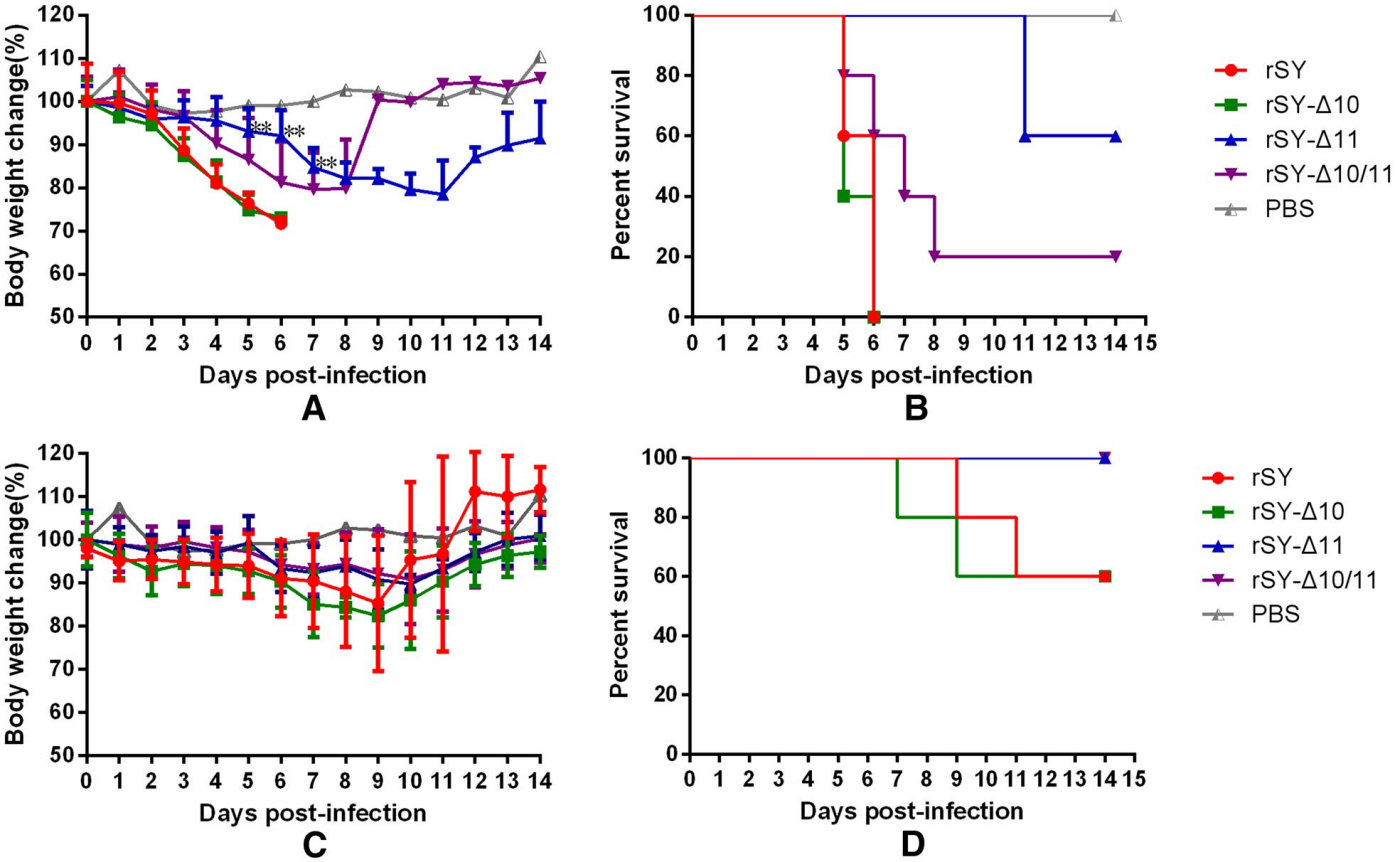
**Pathogenicity of mutant viruses in mice.** Groups of 5 mice were infected intranasally with 10^6^ EID_50_ (**A**, **B**) and 10^4^ EID_50_ (**C**, **D**) of mutant viruses. Mice were monitored daily for weight loss and signs of disease over a 14-day period. Data represents the mean body weight change (%) ± SD. Statistical analysis between the wild-type virus and the mutant viruses was performed using Mann–Whitney test (**p < 0.01).

To compare the virulence of mutant viruses in mice, 6-week-BALB/c mice were injected intranasally with a dose of 10^4^ EID_50_ and 10^6^ EID_50_ of each mutant virus. With 10^6^ EID_50_ infection dosage (Figures 6A and B), the viruses rSY and rSY-Δ10 caused a large decrease in mice body weight and all mice were dead within 7 days. Meanwhile, the rSY-Δ11 and rSY-Δ10/11 viruses caused less decline in body weight and resulted in 60 and 20% survival, respectively. With a 10^4^ EID_50_ infection dosage (Figures 6C and D), mice in rSY and rSY-Δ10 groups had 60% survival, while all mice in rSY-Δ11 and rSY-Δ10/11 groups survived. Dead or euthanized mice at the end of the experiment were taken for virus isolation, and sequencing confirmed that no reversion or unwanted mutation occurred in the isolated viruses.

To further understand the effect of the glycosylation site on virulence in mice. The mice were infected with 10^4^ EID_50_ of each virus. As shown in Table 3, the rSY and rSY-Δ10 viruses replicated efficiently in the lungs on days 3 and 6 post-infection, and replicated less in other organs. By contrast, rSY-Δ10/11 and rSY-Δ11 viruses replicated less efficiently, with the former only being weakly detected in the lungs on day 3 post-infection and the latter absent in any organ on either days 3 and 6 post-infection. These data suggest that the 11N glycosylation site deletion leads to reduced virulence in mice.

**Table 3:** **Distribution of the mutant viruses in mice organs**

Virus	Virus replication in experimentally infected mice [number of virus-positive mice/number tested mice (mean titer ± SD)]											
	Lung		Liver		Spleen		Kidneys		Brain		Heart	
	3 dpi	6 dpi	3 dpi	6 dpi	3 dpi	6 dpi	3 dpi	6 dpi	3 dpi	6 dpi	3 dpi	6 dpi
rSY	3/3 (3.3 ± 1)	3/3 (4.0 ± 0.5)	0/3	0/3	0/3	0/3	0/3	0/3	2/3 (0.8 ± 0)	0/3	0/3	0/3
rSY-Δ10	3/3 (2.9 ± 1.3)	3/3 (4.2 ± 0.6)	0/3	0/3	0/3	1/3 (0.5 ± 0)	2/3 (0.5 ± 0.2)	2/3 (0.8 ± 0.3)	0/3	0/3	2/3 (1 ± 0.5)	1/3 (0.3 ± 0)
rSY-Δ11	0/3	0/3	0/3	0/3	0/3	0/3	0/3	0/3	0/3	0/3	0/3	0/3
rSY-Δ10/11	1/3 (1.5 ± 0)	0/3	0/3	0/3	0/3	0/3	0/3	0/3	0/3	0/3	0/3	0/3

6-week-old BALB/c mice were infected intranasally with 10^4^ EID_50_ of each virus in a 50 μL volume. Organs were collected on days 3 and 6 post-inoculation, and clarified homogenates were titrated for virus infectivity in eggs at initial dilution of 1:10 (lung), 1:2 (other tissues), or undiluted if negative at the lowest dilution.

dpi: days post-infection.

## Discussion

There are a variety of potential glycosylation sites distributed among SY-H5N1 subtype AIV hemagglutinin. Among them, 10/11NNST is highly conserved. A previous study showed that 10/11NNST is an overlapping glycosylation site, the double deletion of which hinders HA_0_ cleavage [17], and that 10N might be a real glycosylation site based on Western blot analysis of viruses mutated from NNST to NPST and NNAA. In this study, three mutant viruses were constructed to delete the 10N, 11N and 10/11NN glycosylation site by substituting NNST with NNAT, NNSA and NNAA, respectively. Although all rescued viruses were confirmed by sequence analysis and were without spontaneous mutations in the first generation, Western blot analysis showed that HA_1_ patterns from the first generations of mutant viruses rSY-Δ11 or rSY-Δ10/11 were not consistent with that from their fifth generation viruses. Further sequence analysis of each generation of the mutant viruses revealed that, since the mutants rSY-Δ11 or rSY-Δ10/11were passaged in chicken embryonic egg or CEF for three generations, the NNSA or NNAA sequences of mutant viruses had been reverted to NNST or NNAT, respectively, which allowed the mutant viruses to form a glycosylation site at 11N again. In a previous study, the fifth generations of mutant viruses (NPST, NNSA, NNAA) were used to determine the HA_1_ patterns by Western blot analysis, this may be the reason that the reverted viruses were used and no change was found between the 11N mutant (NNSA) and the wild-type virus rSY [17]. Since the deletion of 11N glycosylation site reverted quickly, the H5 subtype influenza virus survival may depend on this glycosylation site. This may explain why this overlapping glycosylation site is highly conserved. Our Western blot analysis shows that once the 11-glycosylation site was removed, HA_0_ cleavage was hindered. Only HA_0_ was detectable at the beginning, but this cleavage of HA_0_ still happened during late infection, which resulted in a low virus titer in the early stage of infection in CEF. Also, we found that HA_0_ cleavage correlated with virus growth. When HA_0_ can be cleaved, the virus titer is relative high. We also found that the molecular masses of HA_1_ from the mutant viruses rSY-Δ11 and rSY-Δ10/11 were lower than that of the mutant virus rSY-Δ10 and the wild-type virus rSY. These data indicate that 11N, rather than 10N, is the real glycosylation site, which was consistent with other studies suggesting that NST is more likely to form a glycosylation site among NNST combination [12, 28–30]. We also speculated that steric hindrance may be the main cause of cleavage delay in rSY-Δ11 and rSY-Δ10/11, but this hypothesis remains to be tested.

One of the main functions of stem glycosylation sites is to maintain the structural stability of the virus. Thus, disrupted viral stability due to glycosylation site removal likely accounted for heat and pH sensitivity in rSY-Δ11 and rSY-Δ10/11. However, if 10N could not form a glycosylation site, why is rSY-Δ10 still unstable? It is generally believed that the N-glycosylation site has a fixed amino acid sequence motif: NXS/T(X 〈〉 P). Different X in NXS/T may have different glycosylation stability. In this study, substituting NNST with NNAT did not affect the 10N-glycosylation since 10N is not a glycosylation site, while substituting NST with NAT may affect the 11N glycosylation site [30] or changed the HA structure, which also resulted in lower stability of virus to heat and low-pH.

Head glycosylation of HA may contribute to virulence and antigenicity of influenza viruses, and influenza viruses have variety patterns of head glycosylation [31–34]. However, the role of overlapping stem glycosylation on virulence remains unknown. Although the IPVI of mutant viruses rSY-Δ11 and rSY-Δ10/11 in chickens were lower than that of mutant virus rSY-Δ10 and rSY, both mutant viruses were still highly pathogenic to chickens. In mice, rSY-Δ11 and rSY-Δ10/11 mutants showed a significant reduction in virulence compared to wild-type virus rSY and mutant virus rSY-Δ10, presenting a relative limited and low-titer virus distribution among organs, less body weight loss, and lower mortality. The attenuation of virulence of the mutant viruses in chickens and mice may be attributable to the delay of HA_0_ cleavage [8].

In conclusion, we successfully rescued three modified viruses at the overlapping glycosylation site in the stem of HA, and found that the 11N glycosylation site of SY H5N1 virus was the true glycosylation site, which was critical for HA cleavage and viral virulence. This study facilitates an improved understanding of the role of overlapping glycosylation sites.
